# Challenges and Pitfalls in Human Milk Oligosaccharide Analysis

**DOI:** 10.3390/nu11112684

**Published:** 2019-11-06

**Authors:** Sander S. van Leeuwen

**Affiliations:** Department of Laboratory Medicine, Cluster Human Nutrition & Health, University Medical Center Groningen, Hanzeplein 1, 9713 GZ Groningen, The Netherlands; s.s.van.leeuwen@umcg.nl

**Keywords:** human milk oligosaccharides (hMOS), mass spectrometry, quantitative analysis, lactation, preterm, high pH anion exchange chromatography with pulsed amperometric detection (HPAEC-PAD), Secretor, Lewis

## Abstract

Human milk oligosaccharides have been recognized as an important, functional biomolecule in mothers’ milk. Moreover, these oligosaccharides have been recognized as the third most abundant component of human milk, ranging from 10–15 g/L in mature milk and up to and over 20 g/L reported in colostrum. Initially, health benefits of human milk oligosaccharides were assigned via observational studies on the differences between breastfed and bottle fed infants. Later, pools of milk oligosaccharides were isolated and used in functional studies and in recent years more specific studies into structure–function relationships have identified some advanced roles for milk oligosaccharides in the healthy development of infants. In other research, the levels, diversity, and complexity of human milk oligosaccharides have been studied, showing a wide variation in results. This review gives a critical overview of challenges in the analysis of human milk oligosaccharides. In view of the myriad functions that can be assigned, often to specific structures or classes of structures, it is very relevant to assess the levels of these structures in the human milk correctly, as well as in other biological sample materials. Ultimately, the review makes a case for a comparative, inter-laboratory study on quantitative human milk oligosaccharide analysis in all relevant biological samples.

## 1. Introduction

Human milk is considered the best source of nutrition for growing infants by the World Health Organization (WHO). Exclusive breastfeeding for six months is the optimal way of feeding infants. Thereafter, infants should receive complementary foods with continued breastfeeding up to two years of age or beyond, and it is recommended to exclusively breastfeed for six months. Contrary to cows’ milk and other dairy sources, human milk contains non-lactose oligosaccharides in very high quantities, 10–15 g/L in mature milk and even over 20 g/L in colostrum [1,2]. The complexity of human milk oligosaccharides (hMOS) is also much higher than observed for the oligosaccharides in the milk of most other mammals [3,4,5]. There are many functional properties assigned to hMOS, including prebiotic properties, modulation and maturation of the infant’s immune system, gut (barrier) development, and anti-pathogenic activities [6,7,8,9].

In 1952 Kuhn described the vitamins of human milk [10], which included a novel factor that influenced the growth of bifidobacteria [11,12,13]. Kuhn discovered that the bifidus factor of human milk was a carbohydrate, containing nitrogen. The carbohydrate fraction consisted of oligosaccharides constructed of galactose, glucose, fucose, and *N*-acetylglucosamine [14]. Further studies showed that only GlcNAc containing oligosaccharides that were effective bifidus factors [14]. In the following years, several different structures were elucidated [15,16,17,18,19]. Later studies have identified and annotated over 150 structures [20]. From the identified structures some general observations were made on the biosynthetic rules, and some interpretations could be made on enzymes involved [20,21].

**Figure 1:**
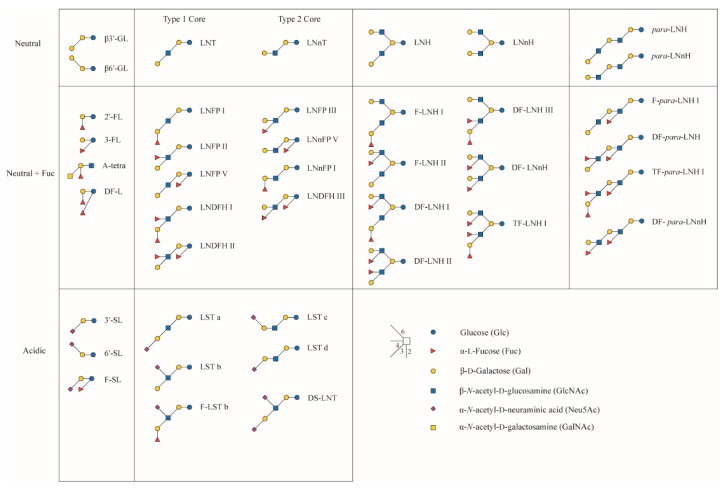
Overview of main human milk oligosaccharides (hMOS) structures for which quantitative data are available. Structures are drawn in the Consortium for Functional Glycomics graphical notation, and a legend for the structural elements is added [48].

Over time, more studies into the benefits of human milk in general, and hMOS specifically, were performed [7,8,22,23,24,25]. These studies show that sometimes there are very specific functions to very specific hMOS. As an example, there is a growing case for di-sialyl-lacto-*N*-tetraose (DS-LNT; Figure 1) in prevention of necrotizing enterocolitis (NEC) in preterm infants [26,27,28,29], with a lesser functionality of 2′-fucosyllactose (2′-FL) [28]. Lewis^x^ bearing structures, particularly lacto-*N*-fucopentaose III (LNFP III; Figure 1), were associated with reduced risk of transfection of HIV from seropositive mothers to their infants [30,31,32], with a suggested dose-dependence. There are many other specific structure–function relationships observed that are very extensively reviewed elsewhere [9,22,33].

With the interest of unravelling the complex functionalities of hMOS, and to elucidate structure–function relationships, it is increasingly important to perform thorough quantitation of specific structures, not only in milk, but also other sample matrices. So far, hMOS have been observed not only in the mother’s milk, but also in the feces of infants up to six months post-partum [34,35,36,37], the blood stream of breastfed infants, and the urine of breastfed infants [38,39]. It has been shown that many hMOS survive the GI tract intact in the earliest phases of lactation and are in ample supply in the feces [36,40,41,42,43]. Over time, the hMOS profile of feces changes and correlations have been made with the development of the microbiome [35,37,41,44]. The hMOS composition of the feces is very variable among individuals. Some infants at one-month age have no detectable levels of hMOS in the feces, while others show a very complete hMOS profile [36,41,44].

Recent studies have also shown hMOS present in the blood stream of pregnant women, [45,46] and in the amniotic fluid [47]. Some of the functions of hMOS are linked to specific structures as described above. To effectively determine whether a structure is present in sufficient concentration to affect its proposed functionality, it is important to understand actual levels of hMOS in all different biological matrices where hMOS may be functional.

This review will focus on the different methods of hMOS analysis in different matrices. An analysis of the different pitfalls of certain approaches will be made. Since most of the work so far has been done on milk, the main portion will discuss the differences in approaches and results on human milk. To place the analytical challenges and result variation into perspective, it is also important to discuss the natural sources of variation in hMOS concentrations.

## 2. Factors Influencing hMOS Composition

Studies found that there are many factors influencing the levels of hMOS observed in milk. The first source of difference that was identified is the time *post partum* of lactation. Colostrum is consistently found to be richer in hMOS than mature milk [1,2]. More detailed studies over the course of lactation have consistently shown that hMOS are not constant over the course of lactation [49,50,51,52,53]. Also, differences were described between mothers delivering term or preterm [54,55]. Other studies, comparing the milk of mothers delivering preterm with term pregnancies, showed no significant differences between the two [56,57]. A recent study showed that there were only minimal differences between term and preterm mothers if you compare the milk in days post-partum [53]. If you consider the time post-menstrual, and thus compare it with the similar infant developmental stage, there are significant differences [53].

Genetic diversity within a population is also a potential source of differences in hMOS composition. One major example is the maternal Secretor- and Lewis-based blood groups. These blood groups are based on genetic variants of the Secretor (α1,2-fucosyltransferase; FucT2) and Lewis (α1,3/4-fucosyltransferase; FucT3) genes [58,59,60,61,62]. These two FucT enzymes are also responsible for the transfer of fucose to hMOS [21,63,64].

**Figure 2:**
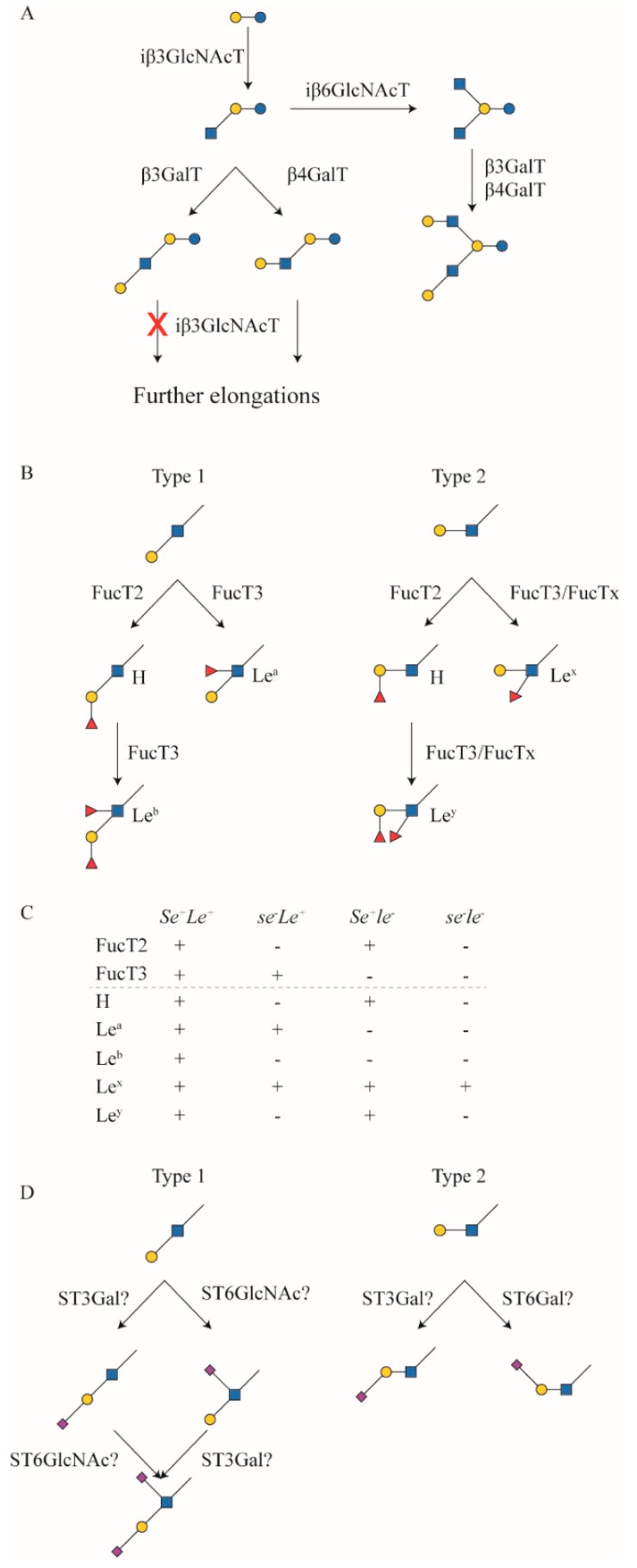
(**A**) Proposed biosynthesis pathways of main core structures adapted from [21] (**B**) biosynthetic pathways of fucosylated epitopes mediated by FucT2 and FucT3; and (**C**) overview of H-antigen and Lewis epitopes present in milk of Secretor (*Se^+^*), non-Secretor (*se^−^*), Lewis positive (*Le^+^*) and negative (*le^−^*) individuals; (**D**) proposed biosynthesis of sialylated structures.

The majority of most populations have both genes functional and active, but in some individuals one or both of the genes contain a single-nucleotide mutation that disables the gene. This results in four so-called milk groups: *Se*^+^*Le*^+^, which has both genes active, *se*^−^*Le*^+^ with a defective FucT2 gene, but a functional FucT3 gene, *Se*^+^*le*^−^ with a functional FucT2 and a SNP in FucT3, and finally the rare *se*^−^*le*^−^ individuals that have defects in both genes (Figure 2). Interestingly, individuals with a defect in the FucT3 gene are still capable of synthesizing hMOS containing (α1–3)-linked fucose, like 3-fucosyllactose (3-FL) and lacto-N-fucopentaose III (LNFP III), but no longer (α1–4)-linked fucose structures, like lacto-N-fucopentaose II (LNFP II; Figure 1). In a few studies on Asian populations, individuals have been observed with neither (α1–3)- or (α1–4)-linked fucose, suggesting that the (α1–3) backup in Lewis-negative individuals is mediated through a specific fucosyltransferase gene, rather than side-activity of several or all (α1–3)-specific fucosyltransferases (FucT4,5,6,7 and 9) [36,65].

Several studies report significant differences in specific, or total hMOS between the milk groups. Most frequently reported are significant increases in lacto-*N*-tetraose (LNT) levels in so-called milk group 4 individuals, that are defective in both FucT2 as well as FucT3. Also, non-secretor individuals, that are defective in FucT2, seem to have higher levels of LNFP II and particularly LNFP III, which was postulated as the effect of lower competition for the precursors LNT and lacto-*N*-neo-tetraose (LNnT) (Figure 1). There is a specific competition between FucT2 and FucT3 for acceptor molecules, with FucT3 blocking activity of FucT2, but not vice versa. LNFP I can still be converted to LNDFH I by activity of FucT3, while LNFP III cannot be converted by FucT2 (Figure 2). Moreover, there were observations that 2’-FL and LNFP I are more abundant in milk group 3 individuals, that are FucT3 deficient, than in milk group 1 participants that have both enzymes active [54,66]. The increased levels of LNFP I in milk group 3 individuals, compared to milk group 1 individuals, could be the result of acceptor competition between FucT2 and FucT3 for the LNT precursor, although LNT is one of the major hMOS. In the case of 2′FL, however, this is clearly not the case, since lactose is present in such excess. Samuel et al. (2019) postulated that it is more likely a result of competition between FucT2 and FucT3 for the GDP-fucose donor substrate, which is limiting [66]. There are also studies indicating that sialyl-lactose is more abundant in non-Secretor individuals. Here, the same statement is true, that lactose is present in excess. However, this does not explain the observed difference in levels of sialylated hMOS between Secretors and non-Secretors [54,66,67,68].

There are also studies reporting significant levels of 2′-FL and LNFP I present in milk from non-Secretor individuals. Some reporting up to 25% remaining 2′-FL [69,70]. In a study by Nakhla et al. (1999), where Secretor and Lewis status was determined by agglutinin screening of saliva samples, it was shown that 2′-FL levels within Secretor mothers milk can be very low [57]. There seemed to be a division between mothers producing 1200–1700 mg/L 2′-FL and a group producing 300–400 mg/L 2′-FL, with few in between. In this study, mothers tested as Lewis-positive showed LNFP II levels between 27–147 mg/L, with one outlier at 1338 mg/L. Lewis-negative mothers showed no detectable levels of LNFP-II. Other studies have seen clear examples of non-Secretors or Lewis-negative individuals where no detectable levels of 2′-FL or LNFP I or LNFP II, respectively, were observed [21,36,56,71,72,73,74]. Interestingly, one paper shows a unique example of a mother that does not produce and 2′-FL during the first nine months of lactation, but then shows a slow increase to normal levels of 2′-FL [75]. In a later study by De Leoz et al. (2012) studying mothers that delivered preterm over time, showed two participants that had no detectable 2′-FL at the first time-point, but had some 2′-FL in one or more of the later time-points. In that same study one other patient had normal levels of 2′-FL detected in the milk, except for one time point in the middle of the studied time-period, where no 2′-FL was detected [55]. These observations indicate that it may not always be correct to make assumptions on Secretor status, based on hMOS composition at one singe time-point.

Finally, strong evidence exists for geographical differences in hMOS composition of the milk. Primarily, differences in Lewis and Secretor status was observed between regions. In Caucasian studies initially ~75%–80% secretor and ~90% Lewis-positive individuals were found. Since there is a large distance between the genes (FucT2 19q13.3, FucT3 19p13.3, >40 M base pairs) on the chromosome the genetic variations are not interdependent [76,77]. This result was shown in 69% of *Se^+^Le^+^*, 20% of *se^−^Le^+^*, 10% of *Se^+^le^−^*, and 1% of *se^−^le^−^* individuals. Recent studies, analyzing milk from different geographical locations showed that there are not only differences between countries (Table 1) [78,79,80], but there can also be differences within a country [36,68]. Not only are the levels of Secretor and Lewis status differentially distributed geographically, but also the general balance of milk oligosaccharides may be different.

## 3. Analysis of hMOS

Glycan analysis is notoriously difficult, for protein glycosylation studies double-blind inter-laboratory examinations have shown that different labs, with different methods will arrive at different results and conclusions. This led to the recommendation to be very specific in methodology when reporting glycan data. So far, it has proven difficult to standardize methods between labs.

Thurl et al. (2017) have evaluated hMOS literature, to find the range and average values of oligosaccharides in human milk [1]. They found 129 eligible studies in total, of which only 21 qualified to be taken into account. Criteria included (i) absolute quantitation of single structures, (ii) milk samples from individual, healthy mothers, (iii) documentation of pregnancy duration, (iv) documentation of lactation days. Studies were excluded when samples were pooled, or lactation time for sampling was not clearly stated. Also, mean values were reported and concentration values should be reported with at least n = 2 samplings per time-point. Most of the excluded studies reported no real quantitation, but relative abundances, reported n = 1 data or did not report on Secretor status when reporting neutral oligosaccharide quantities.

### 3.1. Qualitative Analysis

There are many studies on hMOS that do not look at quantitation of specific hMOS [36,84,85]. Some of these studies do provide general quantitation of total hMOS [86], or relative abundances of hMOS or hMOS classes [55,70,87,88,89]. Many studies also specifically aim to identify novel hMOS [90,91,92,93,94,95], or identify as many hMOS in a milk sample as possible [37,69,87,96]. While these studies provide insight into the complexity of hMOS and are able to assess the inter-individual differences in oligosaccharide composition of milk and other biological samples, they do not provide detailed insight into the absolute quantities, which is most relevant to understand functional properties of hMOS. Many of the qualitative studies have assessed the distribution of so-called milk groups [21,36,64,67,73], based on the presence of activity of FucT2 and FucT3 (Figure 2).

**Figure 3:**
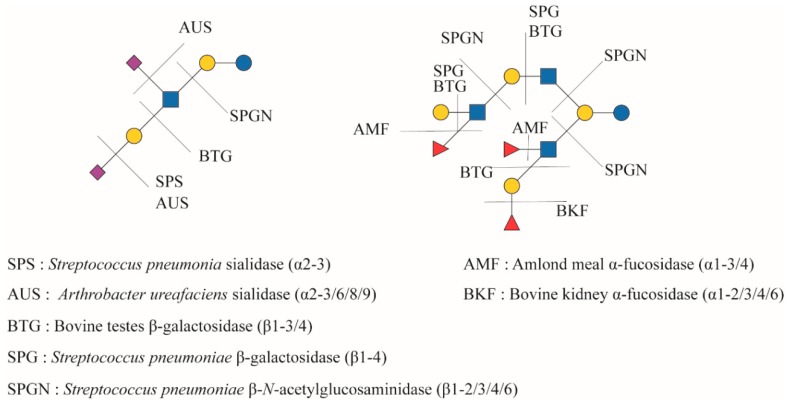
Overview of a suitable set of readily available exoglycosidases most relevant to hMOS analysis [99,100].

In case of shotgun-MS analysis, aiming to identify as many structures as possible in one sample, one of the difficulties is in the identification of the different isomers. For MALDI–TOF-MS fingerprint screening, it is not possible to separate isomers, like LNT and LNnT, or make distinction between isomers of LNFP, since they all have the same monosaccharide composition and mass (Figure 1). Taking specific steps to label Neu5Ac via linkage-specific esterification it is possible to separate Neu5Ac(α2–3) from Neu5Ac(α2–6) in a MALDI–TOF-MS approach [97,98]. Only with fragmentation studies in MS/MS it would be possible to distinguish between isomers with the same linkage type, but at different positions, e.g., LST b from LST c (Figure 1). By coupling the mass spectrometer to a form of liquid chromatography another dimension can be added and elution time adds information about identity, allowing separation between, e.g., LNT and LNnT [69,87,96]. Still, identification of structures for which no standard is available, or the elution time is unknown is difficult. Although MS/MS can help distinguish some structural elements, particularly branch-points, it is still not straightforward to attach exact linkage positions. Some researchers have tackled this challenge by adding sequential exoglycosidase profiling to the analysis, to help identify structures (Figure 3) [87]. It is important to take care in tuning the exoglycosidase assays, since most enzymes have either cross-reactivity at a lower rate for other structural elements or have a minor contamination with another glycosidase enzyme that performs such side-activity at a low rate. Adding too much enzyme or incubating too long may lead to incorrect conclusions. Alternatively, too short assays or employing too little enzyme has the risk of leaving partially undigested peaks left, leading to potential errors in identification as well.

### 3.2. Quantitative Analysis

Kobata et al. (1969) showed a first quantitation of specific oligosaccharides, ranging from 30–700 mg/L for LNT, 0–250 mg/L for LNFP I, and 160–850 mg/L for LNFP II. Difucosylhexaoses were also quantified together between 0 and 750 mg/L [65]. In this study, samples of human milk from nine donors were processed by centrifugation to remove fat, followed by a cold ethanol precipitation overnight to remove a large part of the lactose fraction as well as proteins. Using a size-exclusion chromatography step on Sephadex G25 the hMOS were fractionated further and ultimately analyzed. Quantitation was performed by paper chromatography and chemical staining. This is also one of the first papers showing a difference in hMOS composition between people with a Secretor or a non-Secretor blood group.

In later studies of hMOS, there have been several routes developed for the specific quantitation of hMOS structures in milk. In most cases the hMOS are first isolated from the milk, to avoid interference of highly abundant lactose, lipids and proteins in the milk. These methods are in some cases very elaborate with multiple precipitation, filtering or solid-phase extraction steps [46,65,75,101,102], and in some cases no more than direct fluorescent labeling followed by an online clean-up step in the ultra-high pressure liquid chromatography (UPLC) setup [103,104]. There are also examples of laboratories using a single clean-up step, e.g., acetonitrile precipitation [50,54,105], or solid-phase extraction [56], followed by analysis. Ultimately, the hMOS need to be detected, which can be achieved by adding a label allowing UV or fluorescence detection or by label-free mass spectrometry (MS) based methods. Some studies employed 1D ^1^H NMR spectroscopy to analyze hMOS, either qualitatively [73] or in metabolomics approaches quantitatively [106,107]. It has been noted before that there are significant differences between studies on reported hMOS levels [2,56]. Some studies report values around 4–7 g/L [41,49,108], while other studies report values over 20 g/L [50,54,105]. The differences may be the result of methodological differences, but also due to the natural variation in milk composition. There are multiple factors influencing the amount and composition of hMOS, including blood-group genetics [63,64], time post-partum [53,54,56], geographical influences [36,68,109], and possibly even the gender of the infant [108].

#### 3.2.1. Sample Preparation

Prior to analysis, most studies use several steps of sample preparation. There are many different routes available in literature, but there are some frequently employed steps. The milk contains three main fractions of compounds that potentially interfere with hMOS analysis, i.e., fat, protein, and lactose. In some studies milk salts are also considered an interfering factor and specific steps for salt reduction are taken.

Solid Phase Extraction

There is a multitude of solid-phase materials for extraction of metabolites. In hMOS studies there are two materials used, C18 solid-phase extraction (SPE) resin and porous graphitized carbon (PGC). While some studies employ only graphitized carbon [41,56], there are several other studies that use a combination of both SPE materials in their sample preparation [30,46,68]. Removal of lipids and peptides is achieved by binding to the C18 column, while the oligosaccharides have little affinity to the resin and wash through the column. The PGC is based on complex interactions with graphite and carbohydrates, both involving the hydrophilic character of carbohydrates, as well as hydrophobic stacking [110]. To date, the exact interaction between the resin and oligosaccharides is not well understood. One of the earliest studies already used graphitized carbon to capture hMOS from milk [10]. The graphitized carbon also traps proteins but has such strong affinity that the proteins are not eluted together with the hMOS. To avoid overloading standard PGC-SPE columns with protein, and then losing the lower-affinity hMOS, it might help reducing the protein load beforehand. A first SPE step with C18 can help to reduce the protein load prior to the PGC-SPE step. However, PGC-SPE columns have almost no retaining capacity for monosaccharides and most disaccharides also pass through with minor affinity [110]. This also results in a significant reduction of lactose in the samples, often sufficient to eliminate the interference of lactose in further analysis. One recent study on hMOS showed that 3-FL also eluted from the column very easily and is specifically lost in hMOS isolation using PGC-SPE [111]. This also explains why some papers employing PGC-SPE report lower 3-FL or no 3-FL in their hMOS profiles than others [55,56,69,70,87,112,113]. So far, a few studies have covered this by alternative quantitation of 3-FL separately from the total pool of hMOS that were isolated with PGC-SPE [41,111]. A large study by McGuire et al. (2017) used two consecutive SPE steps using C18 and PGC in sequence applying fluorescent labeling and HPLC analysis and still report 3-FL [68], but is significantly lower quantities than another study using direct fluorescent labeling of untreated milk followed by HPLC analysis [49,53]. In a recent study, by Kunz et al. (2017), graphitized carbon SPE was used, followed by quantitative high pH anion exchange chromatography with pulsed amperometric detection (HPAEC-PAD) analysis. Here 3-FL was reported to co-elute with LNDFH I, however in the non-Secretor sub-population of this study no 3-FL was reported, indicating that probably all 3-FL was lost during SPE clean-up of the samples. It should be noted that LNDFH I is a structure containing an (α1–2)-linked fucose epitope and is thus absent in non-Secretor samples [56].

These observations raise the question whether there are other specific losses of structures when using either PGC-SPE or C18-SPE as a sample preparation step.

Centrifugal Fat Separation

A large amount of studies on hMOS start with removing or reducing the lipid content of the milk by centrifugation [44,57,73,81,107,108,114,115,116,117]. In some studies, the fat layer on top of the sample is scooped off the milk, in other studies the clear liquid is pipetted into a new vial, carefully avoiding transferral of fat to the new vial. Some studies follow this step with SPE to further remove fat [73,117], while other studies consider a significant reduction of fat sufficient. Some researchers have chosen to start with a larger sample volume than required, diluting the sample and then taking a fixed exact amount of clear liquid under the fat-layer after centrifugation. This may be the most precise method for defatting and if carefully executed has little risk for specific losses of hMOS or large variations in residual fat levels. Using a plate-rotor in the centrifuge allows this step to be incorporated in 96-wells plate high-throughput procedures [118].

Liquid Extraction

Some studies employ a traditional Folch extraction [119], with chloroform:methanol (2:1) to remove fat, and incidentally reduce the protein load of the sample, extracting denatured protein in the chloroform phase [4,57,101,113,120,121,122]. Such liquid extractions are more effective in removing the fat layer than scooping the fat layer off centrifuged samples but are quite labor intensive to perform. When separating the chloroform layer from the hydrophilic layer it is important to take all the chloroform or be left with fat. If the researcher removes part of the water layer, together with the chloroform layer, then part of the hMOS will be lost as well. In practice, this approach often requires centrifugation to resolve the two phases properly. Although this approach is labor intensive and not very suited to high-throughput approaches or handling many samples, there have been studies with extensive sample sizes employing a Folch extraction [30].

Ultrafiltration

To avoid interference from proteins in the analysis of hMOS, an ultrafiltration step can also be used. This approach involves centrifugal filters with a MW cut-off, most frequently 10 kDa filters are used [78,81,108], but some studies employ 3 kDa [107,116] or 30 kDa [38,71]. Tonon et al. (2019) verified the efficiency of the filters and found no significant losses of hMOS for which they had quantitative references [81]. One of the risks in using centrifugal filters is clogging of the filter with solids, which is mostly a practical problem, which can likely be avoided by preceding the ultrafiltration step with centrifugal removal of fat, in which solids will mostly precipitate to the bottom of the vial.

Organic Solvent Precipitation

Another method of protein removal involves the use of organic solvents to precipitate the proteins. A series of publications employed 1:1 mixing of milk with acetonitrile to precipitate the protein. The clear liquid was used to analyze hMOS directly in an HPAEC-PAD based method of analysis [51,54,79,105,123,124]. Other studies, like the early approaches of Kobata et al. (1969) [65] used ice-cold ethanol precipitation to remove protein. An added advantage of the ethanol approach is the partial lactose precipitation. Most oligosaccharides remain in solution in 67% ethanol. The most studied hMOS do not appear to co-precipitate in this approach [118], but there has not been a detailed study of the precipitate to determine if some of the larger size hMOS might be lost in this step. A third option for precipitation with organic solvents involves acetone [5]. Our lab has recently shown in N-glycan studies, however, that acetone has potential for precipitation of larger oligosaccharides and particularly sialylated glycans, while smaller neutral glycans are less affected [125]. It might be a limited problem in the size-range usually encountered in hMOS, but it would be recommendable to verify the method.

One major advantage of organic-solvent precipitation steps is that they are compatible with high-throughput approaches and also applicable in 96-wells formats. One example is a very rapid method, combining centrifugal defatting with ethanol precipitation of proteins in a 96-wells format [118]. For really large sample sets, which may be required to answer the currently most pressing questions on diversity and functionality, it may be crucial to adopt a rapid 96-wells format approach. However, such a high-throughput approach should be evaluated to ensure it does not suffer from losses of specific hMOS, as was observed for PGC-SPE.

Gel Filtration Chromatography

In some of the earliest hMOS studies gel-permeation chromatography was used [65,126], mostly to separate the hMOS from the disaccharide lactose. Kobata et al. (1969) commonly used Sephadex G25 to achieve size-based separation of hMOS, and then studying the different size fractions separately. Other studies pool all the fractions that are shown to be lactose free and analyze the pool [5,127,128]. Other column types have also been employed, e.g., Toyopearl HW50 [71,129] or Biogel P2 [52,121,122], but the principle of size based separation is the same. One of the challenges of such a gel filtration chromatography is to correctly isolate the broad peaks. There tends to be overlap between size-species. Particularly lactose will overlap with tri- and tetrasaccharide-peaks. Eliminating all fractions containing lactose will achieve a lactose-free hMOS pool but might incur specific losses of the smaller hMOS. Another disadvantage of the gel filtration approach lies in the time limitations; gel filtration columns work at low flow-rates and run-times can take between 1.5 and 32 h per sample [52,71,121,122,129]. This limitation is prohibitive towards larger sample sets and high-throughput analyses.

#### 3.2.2. Analytical Methods

Analysis of the hMOS usually makes use of a separation technique, coupled to a form of detection, that is capable of a semi-linear response curve for increasing analyte concentrations. Separation is achieved by either liquid chromatography on hydrophilic-interaction (HILIC) or anion-exchange chromatography (AEC) based column, or by capillary electrophoresis, which is based on the relation between hydrodynamic volume of the analyte and the charge of the analyte. In case of capillary electrophoresis, usually a multiply charged label (8-aminopyrene-1,3,6-trisulfonic acid; APTS) is used, to ensure that all oligosaccharides carry a negative charge. The label of choice is also usually fluorescent, to achieve sensitive detection.

To detect the oligosaccharides after separation there is a choice between labelled (fluorescent or UV active labels covalently coupled to the oligosaccharides) and label-free detection, e.g., refractive index (RI), evaporative light scattering detection (ELSD), pulsed-amperometric detection (PAD), and mass spectrometry (MS). Since RI has limited sensitivity and is limited to isocratic separation conditions, this detection strategy is not feasible. Similarly, ELSD will suffer from limited compatibility with common oligosaccharide separation gradients. This means that label-free detection is essentially limited to PAD and MS. Some studies, however, employ UV detection of unlabeled sialylated structures, but the sensitivity is limited and some of the most abundant neutral oligosaccharides cannot be detected in this way (e.g., 2’-FL and 3-FL).

Liquid Chromatography

An attractive way to overcome this problem of detection, particularly for neutral oligosaccharides, is the addition of a fluorescent label via reductive amination. Each oligosaccharide molecule acquires one fluorescent label on the reducing end, most frequently 2-aminobenzamide (2-AB) or 2-aminobenzoic acid (2-AA), but 2-aminoacridone (2-AMAC) has also been used. Since each molecule carries a single fluorescent label, there is in essence an equal and linear response for each structure [130]. This eliminates response factors in the detection, although differences in labeling efficiency between hMOS seem to exist, resulting in calibration curves for standards with different slopes [104]. The highest and lowest curve slopes in the study by Austin and Bénet showed a factor ~1.3 difference. Maltotriose had a slope close to the means of all slopes, which makes this a good general calibrant, also suitable for structures of which standards are not available.

There have also been some alternative labeling approaches to achieve detection, including per-benzoylation of isolated oligosaccharides [75], to increase the UV absorbance of hMOS, as well as allowing C18 chromatography separation. Care must be taken to avoid under-benzoylation and non-homogeneous labeling efficiency is a potential risk in such an approach. Several quantitative studies have employed a UV-active label 1-phenyl-3-methyl-5-pyranozolone (PMP) to label the reducing end of hMOS and achieving sensitive detection [4,101,121,122].

Capillary Electrophoresis

Separation of glycan structures by capillary electrophoresis (CE) has been established for protein glycans [131]. To ensure that all glycans carry a negative charge, to make them active in electrophoresis, it is common to attach a charges label, usually 8-Aminopyrene-1,3,6-trisulfonic acid, but also 2-AA and 2-AMAC are suitable for this purpose [124]. One study has used CE on sialylated glycans [40,82,117], and detected them by the UV-absorption of the N-acetyl groups [114].

One of the main challenges in CE is that the migration-rate through the column is proportional to the size—charge ratio. This means that structural isomers will co-elute, e.g., 2′-FL and 3-FL; LNT and LNnT, will co-elute. Therefore, studies employing CE can only report quantities of these structures together. In some cases, the structural isomers have a slightly different hydrodynamic volume, and are slightly separated on the column. Using proper settings allows sufficient separation to quantify these structures separately. Galeotti et al. (2014) were not able to separate LNFP II and LNFP III, but both were separated sufficiently from LNFP I. In the same study 2′-FL and 3-FL were separated, but 3′-SL and 6′-SL were not [124]. Another study, by Olivares et al. (2015), was able to separate LNFP II, but LNFP I and III co-eluted, similarly, co-elution was observed for 2′-FL with 3-FL, LNT with LNnT and 3′-SL with 6′-SL [117]. Although other separation techniques also have occasional co-eluting species, the level of co-elution on CE is much more prominent. This is one of the major drawbacks of CE approaches. The advantages of CE are the easy coupling to mass spectrometry, rapid analysis times, and very sharp peaks.

HPAEC-PAD

Finally, there is another chromatographic method that requires no labeling. Here, oligosaccharides are separated in a high pH chromatography system. At the high pH employed, the hydroxyl groups are deprotonated and the difference in pK_a_ of the various hydroxyls plays an important role in the separation properties on an anion-exchange column (HPAEC) [132,133]. An added advantage of the high-pH based deprotonation of hydroxyls is the possibility to detect the oligosaccharides with pulsed-amperometric detection (PAD). One of the major disadvantages is that the differential deprotonation of oligosaccharides also induces a vast range of response factors on the detector [134,135]. Different studies on hMOS have reported vastly different response factor ranges, but this may be the result of different chromatography gradients, as the response factor is strongly pH dependent and a difference in elution gradient may induce a difference of pH when a specific oligosaccharide is eluted [50,54,134,135]. This means that each lab will have to determine response factors themselves, and due to minor changes in buffers between runs each new analysis requires a new response-factor determination to be precise in results. Another disadvantage of the PAD-detection is the relatively limited linearity in detector response [132,133]. One way to cover this, is by injecting samples in two different concentrations to quantify major and medium compounds separately, Kunz et al. (2017) have alternatively employed a quadratic calibration curve [56]. This overcomes the limitations in linearity, but at higher concentrations the standard deviation in concentration derived from a certain integration increases.

These limitations notwithstanding, there are several studies using HPAEC-PAD and reaching quantitative results with good repeatability within their own lab. Moreover, the quantities reported in some of the studies are well within reach of most other reports [1,56,78]. Other studies using HPAEC-PAD, employing a 3-point calibration curve for some references, report relatively high values [50,54,105]. A comparison between methods from one of these studies showed rather high differences between analysis by HPAEC-PAD, LC coupled to a laser-induced fluorescence (LIF) detector or LC–MS approaches [105], although the HPAEC-PAD showed the lowest total hMOS concentrations in most cases. A later study using CE-LIF also reported high values for the same samples [124]. All studies on this sample set employed a protein precipitation step with 50% acetonitrile as sole extraction step. For fluorescence detection and MS analysis the samples were labelled via reductive amination, and for HPAEC-PAD no labeling was employed. All reported values with the different studies are well above generally reported ranges [1]. Whether this is specific to this set of samples from Italy, or an experimental bias is unknown.

NMR Spectroscopy

A more rare method is NMR based metabolomics, which does not add a separating technique, but instead focuses on specific combinations of bins in the NMR spectrum indicative of specific structural elements [106,107]. A more detailed structural-reporter-group concept has also been developed in the past also for N- and O-glycans [136,137]. In our lab we found that specific structures, with the exception of 2′-FL, were not readily detected and quantified by NMR spectroscopy of an isolated hMOS pool [73]. Most similar structures overlap, e.g., all hMOS bearing a Lewis^b^ epitope showed overlapping structural-reporter-group signals. The sole exception was 2′-FL, for which a specific structural-reporter signal was available. General structural identities can be analyzed quantitatively, and distinction between the different Lewis epitopes, H-antigen and sialic acid elements could be made effectively [36]. In metabolomics studies, usually no clean-up steps are applied or as few as possible. Minimal sample treatment avoids specific losses of structures, such as loss of 3-FL by graphitized carbon SPE. However, many other components of the biological sample will render overlapping peaks, increasing the difficulty in selectivity and quantitation of specific hMOS structure groups.

Mass Spectrometry-Based Methods

In mass spectrometry there is a difference in discovery driven “shotgun” approaches, where quantitation and repeatability of results are generally limited, while identification of new biomolecules is very high [138]. In targeted analyses the repeatability and quality of quantitation is much improved. However, there is still the question of reproducibility, i.e., the same sample analyzed by another operator or another lab on a similar system, reaching the same result [139]. The challenges of mass spectrometry based quantitation are well described for proteomics approaches [138,139], but not exhaustively yet for carbohydrate analyses. However, some basic principles apply to any MS approach. There is a difference between mass spectrometer sensitivity and the limit of detection (LOD). Sensitivity of the machine is determined by the minimum amount of pure analyte required to elicit a response on the detector. The LOD is also influenced by the signal-to-noise level achieved in the context of the sample matrix and elution conditions. In transition monitoring methods, increased amount of transitions increases the noise level, and therefore negatively influences the LOD [139]. The dynamic and linear ranges of machines are also relevant; if a mass detector gets saturated for main compounds, before minor compounds of interest can be effectively quantified, the dynamic range is too limited. Alternatively, two different concentrations can be measured to cover both major, and minor compounds. In general, in-beam instruments have a better dynamic range than ion-trap instruments, due to increases in noise levels in longer trapping times. Furthermore, calibration curves are usually determined using pure compounds dissolved in pure solvents, while real biological samples have matrix contaminants that can induce ion-suppression [140]. Co-eluting compounds and background therefore influences ionization efficiency (ionization suppression and ionization enhancement) and dynamic range of the analytes of interest. In-spiking of isotope-labelled standards for quantitation is still the golden standard, but some advancements in machines, methods and software have improved label-free quantitation significantly in the last decade [138]. If proper steps are taken, good quantitation with MS is possible, also without isotope-labeling, reaching accuracies within 15% deviation [111,141,142]. Many of the most recent LC-MS based quantitative approaches achieve hMOS values comparable with most other studies [41,111,141].

## 4. Conclusions

Taking all these factors into account, it is very difficult to compare different studies. As established there are great differences in methods employed to analyze hMOS. Some studies use a very elaborate sample treatment prior to analysis and quantitation of hMOS, while others have very simple approaches. It is important to realize that most sample treatment steps have a risk of specific losses of some hMOS structures or introduction of methodological biases. As shown by Xu et al. (2017), the application of porous graphitized carbon SPE columns led to the specific loss of 3-FL [111]. One study assessed specifically that no bias in structures was induced by ultrafiltration [78]. However, most other separation techniques have not been extensively assayed for specific losses of hMOS.

It is also important to note that some analytical approaches are essentially better suited to quantitative analysis than other. Although the difficulties of HPAEC-PAD quantitation can be overcome with very good reference calibration curves to determine response factors and to cover lack of linearity in dose-response with quadratic curves, detection of fluorescently labelled oligosaccharides with a large linear response range is essentially better suited for quantitative analysis. While MS has good potential for long linear ranges, there is the problem of response factors to contend with. Essentially, only oligosaccharides for which a reference standard is available can be effectively quantified. Additionally, steps have to be taken to assess possible matrix associated loss of sensitivity and ion-suppression/enhancement.

Even when using fluorescent labeling via reductive amination of free oligosaccharides, and as few sample treatment steps as possible, there is still a difference in labeling efficiency to be taken into account [104]. In this case, however, the response factors for different oligosaccharides seems to be limited. The level of accuracy of the method using an internal maltotriose standard rather than true hMOS references still achieved spike-in recoveries within acceptable levels of deviation [104].

To close, I would like to repeat the recommendation by Thurl et al. (2017), that a double-blind multi-center study of hMOS analysis would be very beneficial to assess the true levels of hMOS in human milk [1]. This will help assess how big the variation in hMOS composition is between milk groups, between individuals within a geographical region, but also between the regions of the world. Moreover, it would be beneficial to also assess efficacy of the different analytical approaches for analyses in blood, urine, feces, and amniotic fluid. The next steps forward in hMOS research would strongly benefit from solid quantitative data in all relevant biological matrices.

## Figures and Tables

**Table 1:** Overview of geographical distributions of Secretor (Se) and Lewis (Le) occurrence.

Country	n=	Se (%)	Le (%)	Reference
Brazil	77	88	88	[81]
Burkina Faso	53	76	87	[79]
Chile	44	84	80	[78]
China	32	78	91	[78]
China	540	79	nd	[49]
China Sohhot	30	80	94	[82]
Ethiopia Rur	40	65	nd	[68]
Ethiopia Urb	40	78	nd	[68]
France	22	91	68	[78]
France	83	87	89	[66]
Gambia Rur	40	65	nd	[68]
Gambia Urb	40	85	nd	[68]
Germany	18	83	100	[78]
Germany	50	78	89	[64]
Germany	30	83	90	[71]
Ghana	40	68	nd	[68]
Italy	63	66	84	[54]
Italy	29	86	86	[78]
Italy–Sicily	50	77	97	[79]
Kenya	42	81	nd	[68]
Malaysia	20	65	90	* Unpubl
Mexico	316	100	74	[83]
Mexico	156	100	83	[78]
Netherlands	32	72	88	[73]
Netherlands	121	73	nd	[41]
Peru	43	98	nd	[68]
Philippines	22	46	96	[78]
Portugal	95	66	86	[66]
Romania	40	90	93	[66]
Singapore	26	72	96	[78]
Spain	32	66	81	[56]
Spain	41	76	nd	[68]
Sweden	24	79	nd	[68]
Sweden	40	90	95	[66]
Switzerland	61	79	94	[53]
USA	79	68	90	[78]
USA Calif	19	95	nd	[68]
USA Wash	41	68	nd	[68]
Vietnam	101	60	87	[36]
Ho Chi Minh	18	72	89	[36]
Ha Long Bay	20	80	90	[36]
Hanoi	21	33	86	[36]
Phu Tho	22	64	91	[36]
Tien Giang	20	55	80	[36]

Some regions have been analyzed by multiple studies. Rur indicates rural environment and Urb indicates urban environment, nd indicates that no data were available. * indicates an unpublished sample set from our lab.
